# A critique of measurement of defective insulin secretion and insulin sensitivity as a precision approach to gestational diabetes

**DOI:** 10.1007/s00125-024-06334-x

**Published:** 2024-12-02

**Authors:** Danielle L. Jones, Laura C. Kusinski, Clare Gillies, Claire L. Meek

**Affiliations:** 1 Institute of Metabolic Science Metabolic Research Laboratories, University of Cambridge, Cambridge, UK; 2 Leicester Diabetes Centre, Leicester General Hospital, University of Leicester, Leicester, UK; 3 University Hospitals Leicester NHS Trust, Leicester General Hospital, Gwendoline Road, Leicester, UK

**Keywords:** Diagnosis, Gestational diabetes, Insulin resistance, Insulin secretion, Oral glucose tolerance test, Pregnancy

## Abstract

**Aims/hypothesis:**

Precision medicine approaches to gestational diabetes mellitus (GDM) have categorised patients according to disease pathophysiology (insulin resistance, insulin insufficiency or both), and demonstrated associations with clinical outcomes. We aimed to assess whether using enhanced processing to determine indices of insulin secretion and sensitivity is analytically robust, reproducible in a different population, and useful diagnostically and prognostically in clinical practice.

**Methods:**

A total of 1308 pregnant women with one or more risk factors for GDM who underwent a 75 g OGTT at one of nine hospital sites were recruited to this observational study. Specimens were collected for determination of glucose levels using standard and enhanced procedures, HbA_1c_ and insulin analysis. GDM diagnosis and management followed National Institute for Health and Care Excellence guidance. We categorised women into pathophysiological subtypes: insulin-resistant GDM (HOMA2-S < 25th centile of the population with normal glucose tolerance [NGT]), insulin-insufficient GDM (HOMA2-B < 25th centile), both or neither. We assessed associations with pregnancy outcomes using logistic regression.

**Results:**

Using enhanced specimen handling, 1027/1308 (78.5%) women had NGT, with 281/1308 (21.5%) being classified as having GDM. Of this group, 135/281 (48.0%) had insulin-resistant GDM, 73/281 (26.0%) had insulin-insufficient GDM and 2/281 (0.7%) had both insulin-resistant and insulin-insufficient GDM. Unexpectedly, 71 patients (25.3%) had GDM with both HOMA2-S and HOMA2-B ≥ 25th centile (GDM-neither). This novel subgroup appeared to be relatively insulin-sensitive in the fasting state but developed marked post-load hyperglycaemia and hyperinsulinaemia, suggesting an isolated postprandial defect in insulin sensitivity that was not captured by HOMA2-B or HOMA2-S. Women within most GDM subgroups had comparable pregnancy outcomes to those of normoglycaemic women, and HOMA2-B and HOMA2-S were weak predictors of pregnancy outcomes. Maternal BMI predicted a similar number of outcomes to HOMA2-S, suggesting that there was no additional predictive value in adding HOMA2-S. Similar findings were obtained when using different indices and standard specimen handling techniques.

**Conclusions/interpretation:**

Precision categorisation of GDM using HOMA2-S and HOMA2-B does not provide useful diagnostic or prognostic information, but did distinguish a novel subgroup of patients with GDM, characterised by an isolated postprandial defect in insulin sensitivity.

**Graphical Abstract:**

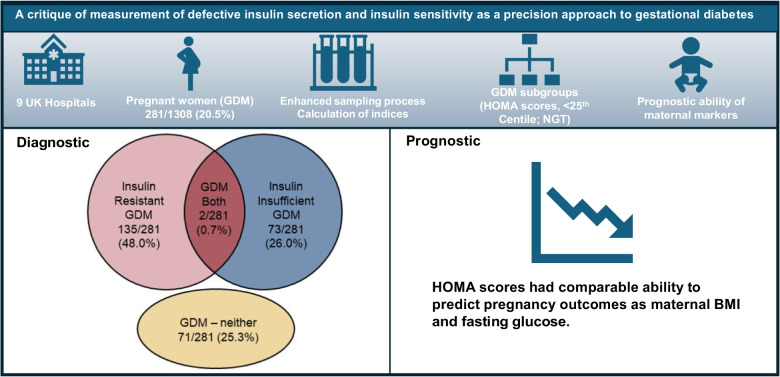

**Supplementary Information:**

The online version of this article (10.1007/s00125-024-06334-x) contains peer-reviewed but unedited supplementary material.

## Introduction


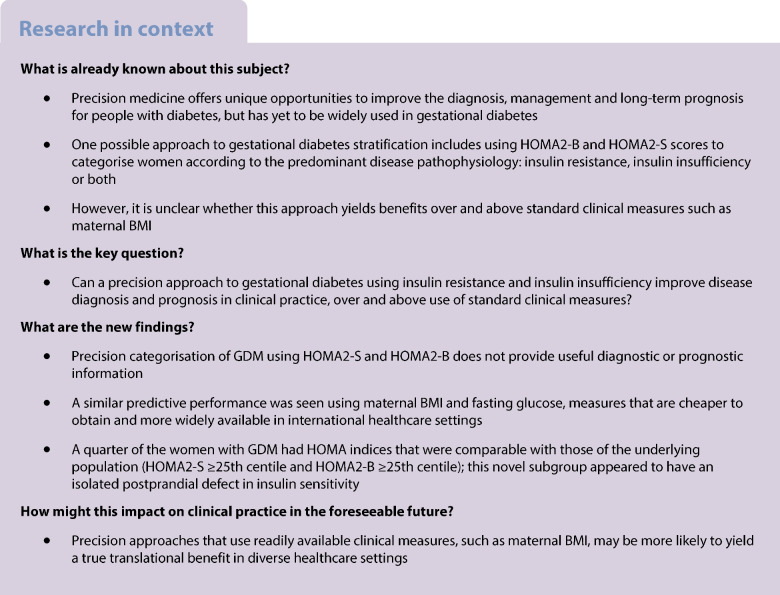


Precision medicine offers unique opportunities to revolutionise the diagnosis, management and long-term outlook for people with diabetes, but has yet to be widely used in gestational diabetes mellitus (GDM) [1]. The recent consensus report by Tobias et al as part of the ADA/EASD Precision Medicine in Diabetes Initiative outlined opportunities for precision approaches to improve GDM prevention, diagnosis, treatment and prognosis, suggesting that these should be key areas for focus [1]. However, the value of a precision approach to GDM has not yet been fully realised in clinical practice [2, 3].

GDM, which is considered a maladaptation of maternal metabolism to meet the demands of pregnancy, has been attributed to disordered insulin sensitivity, insulin secretion or both [4]. Using this pathophysiological categorisation, it has been reported that women with predominantly insulin-resistant GDM have higher rates of pregnancy complications, such as preterm delivery, unplanned Caesarean delivery, neonatal hypoglycaemia and admission to the neonatal intensive care unit [5]. Conversely, women with GDM due to defective insulin secretion in pregnancy appear to have comparable pregnancy outcomes to those of women with normal glucose tolerance (NGT), but have persistent beta cell deficits postpartum [4, 6]. At present, GDM is treated as a homogeneous disease with a common treatment strategy. If successful, a precision approach to GDM could potentially allow disease pathophysiology to inform management, allowing earlier allocation to successful treatments that could improve outcomes for mother and neonate.

Classifying GDM into subtypes is challenging due to the complexities of measuring the dynamic physiological processes of pregnancy. Use of euglycaemic glucose clamps is the reference standard, but this is not feasible for widespread use [7]. Many studies in pregnancy have used mathematical indices, such as HOMA scores, Matsuda and Stumvoll indices, which use glucose and insulin concentrations to assess insulin resistance or secretion [8, 9]. Some of these indices have been validated against euglycaemic clamps in small groups of pregnant women [10, 11], but the insulin or C-peptide analysis required for these indices is not available in all clinical settings. Differences in pre-analytical specimen handling introduce inaccuracies, and therefore larger clinical cohort assessments are needed before wider clinical application.

This study aimed to evaluate the clinical utility of a precision medicine approach to GDM, using indices of insulin secretion and sensitivity to categorise women into GDM subgroups, and assessing associations with pregnancy outcomes. In concordance with the recent consensus statement [1], we considered that a successful precision approach to GDM could offer improved diagnostic or prognostic value. We therefore hypothesised that this precision medicine approach could categorise all women with GDM into discrete pathophysiological subgroups (diagnostic value) with distinct clinical outcomes (prognostic value) using tests that were accurate and reproducible in clinical care.

## Methods

### Study design and participants

We recruited 1308 women with at least one risk factor for GDM [12] to the OPHELIA study (Observational study for Pregnancy Hyperglycaemia, Endocrine causes, Lipids, Insulin and Autoimmunity) at nine UK hospital sites (REC 18/LO/0477; research registry number 5528) between October 2018 and March 2023. The recruiting sites were Hinchingbrooke Hospital, Huntingdon; Queen Charlotte’s and Chelsea Hospital, London; Lister Hospital, Stevenage; Colchester General Hospital, Colchester; Norfolk and Norwich University Hospital, Norwich; Peterborough City Hospital, Peterborough; Salisbury District Hospital, Salisbury; Croydon University Hospital, Croydon; Ipswich Hospital, Ipswich. Study sites were selected to obtain a broad representation of the larger population in terms of ethnicity, age and BMI. A detailed assessment of precise socioeconomic inequalities across England was beyond the scope of this study. Pregnant women were recruited prior to attending for a 75 g OGTT. Eligible women had a singleton pregnancy with no evidence of severe congenital anomalies. Exclusions included severe anaemia, pre-existing diabetes, or medications (e.g. metformin, corticosteroids) that could affect the OGTT results. The study was conducted in accordance with the Declaration of Helsinki and good clinical practice. All women provided written informed consent prior to study enrolment. GDM diagnosis and management followed NICE guidance at all sites [12].

### Biochemistry

After an 8–12 h overnight fast, pregnant women underwent a 2 h OGTT. Venous blood samples were obtained at 0 and 120 min after ingestion of a standard 75 g glucose drink. Blood samples were taken for determination of glucose levels and HbA_1c_; these were processed and analysed at local hospital laboratories at each study site using local protocols and methods (predominantly glucose oxidase methods for glucose and HPLC (Tosoh analyser, Tosoh Bioscience, Japan) for HbA_1c_, aligned with the International Federation of Clinical Chemistry and Laboratory Medicine (IFCC).

As glucose concentrations are influenced by pre-analytical sample handling conditions, additional blood samples were taken and processed using an enhanced pathway. Samples were cooled to 4°C using ice or a cool pack, centrifuged at 2500 rpm (1500–2000 *g*) for 10 min, aliquoted and rapidly frozen for later batch analysis. For the enhanced specimen handling pathway, we performed a second glucose analysis on the frozen samples using a hexokinase method, which is generally considered more accurate than glucose oxidase methods. Glucose determination for the enhanced processing pathway used a hexokinase method (Siemens Dimension, Siemens, Germany), which was performed strictly according to the manufacturer’s guidelines (issue date 1 April 2019). Samples were defrosted and thoroughly mixed immediately prior to analysis. This assay has a working range of 0–27.8 mmol/l (0–500 mg/dl), a lower limit of detection of 0.056 mmol/l (1 mg/dl) and high degrees of intra-assay precision (CV 1.2–1.6% at the concentrations range used in this study). Samples that were visibly lipaemic or haemolysed were not analysed. In our laboratory, the inter-assay precision was 2.5–3.6%, and calibrators were used immediately before running the samples. Insulin concentrations were determined using a Liaison analyser (DiaSorin, Italy). This method has a range of 20–3470 pmol/l and an intra-assay CV of 5.0–6.0% across the analytical range. The insulin standard is aligned with the WHO first international reference preparation (66/304).

### Data collection

Self-reported data were collected for medical and obstetric history, symptoms and risk factors for GDM, including ethnicity, family history of diabetes in first-degree relatives, previous macrosomic infants (>4.5 kg), previous GDM, and history of related conditions such as polycystic ovary syndrome. If this information was unknown, data were retrieved from the hospital’s electronic systems, as well as weight and blood pressure measured at antenatal visits and pregnancy outcome data. GDM diagnosis was based upon NICE criteria ( 5.6 mmol/l [≥101 mg/dl] at 0 min; ≥7.8 mmol/l [140 mg/dl] at 120 min) [12].

### Precision categorisation into subgroups

Using glucose and insulin concentrations, we calculated indices of insulin sensitivity (Matsuda, HOMA2-S and HOMA2-IR; see electronic supplementary material [ESM] Table 1) and indices of insulin secretion (HOMA2-B, disposition and Stumvoll indices; ESM Table 1) [7, 13, 14]. The HOMA2 indices [15] were calculated using the complex model provided at https://www.rdm.ox.ac.uk/about/our-clinical-facilities-and-units/DTU/software/homa. HOMA2-IR models insulin resistance, whereas HOMA-S is a variant of the HOMA model that specifically estimates insulin sensitivity. We planned a priori to categorise women into groups in line with the method described by Powe et al [4]. Women with NGT had normal glucose levels on enhanced specimen processing, which fell below the thresholds for GDM diagnosis. Women with insulin-resistant GDM (GDM-IR) had glucose levels consistent with GDM on enhanced specimen processing, with HOMA2-S < 25th percentile and HOMA2-B ≥ 25th percentile for the population with NGT. Women with insulin-insufficient GDM (GDM-IS) had glucose levels consistent with GDM on enhanced specimen processing, with HOMA2-B < 25th percentile and HOMA2-S ≥ 25th percentile for the population with NGT. Women with both insulin-resistant and insulin-insufficient GDM (GDM-both) had glucose levels consistent with GDM on enhanced specimen processing, with HOMA2-B < 25th percentile and HOMA2-S < 25th percentile for the population with NGT. Women with GDM with no evidence of insulin resistance or insulin insufficiency (GDM-neither) had glucose levels consistent with GDM on enhanced specimen processing, with HOMA2-B ≥ 25th percentile and HOMA2-S ≥ 25th percentile for the population with NGT.

### Definition of outcomes

We included pregnancy outcomes as pre-specified in the research registry protocol for this study (https://www.researchregistry.com; study number research registry 5528). We adhered to the core outcome set for studies on prevention and treatment of GDM [16]. Neonatal hypoglycaemia was defined as a plasma glucose level of less than 2.6 mmol/l (47 mg/dl) within the first 24 h of life. Neonatal intensive care unit admission included infants admitted for a period of at least 24 h within the first 28 days of life, with the criteria for admission being similar between the participating centres. A preterm delivery was considered as delivery at 37 weeks’ gestation or earlier. Gestational age was determined using the estimated delivery date obtained at dating ultrasound scans.

### Statistical analysis

Values for baseline characteristics, concentrations of glucose and insulin, and indices of insulin sensitivity/resistance and secretion are presented as mean ± SD, *n* (%) and median (range) as appropriate. Using an unadjusted logistic regression model, we assessed associations of HOMA2-S and HOMA2-B, maternal BMI and fasting glucose with pregnancy outcomes. This model was then adjusted for GDM diagnosis and maternal age. Neonatal outcomes were adjusted for gestational age at birth in both models. The INTERGROWTH [17] and GROW [18] growth standards were used to generate centiles for categorisation of infants as large for gestational age (LGA). Areas under the receiver operating characteristic curve (AUROC) and areas under the precision-recall curve (AUPRC) were calculated to assess the ability of HOMA2-S, HOMA2-B, maternal BMI and fasting glucose to predict pregnancy outcomes. Differences in AUROC were compared using the χ^2^ test. AUPRC values are presented with the percentage of positive cases for each outcome to aid interpretation. Missing data were not imputed. Our sample size provided 80% power at an α value of 5% to detect a 20% difference in rates of LGA between women in the GDM-IS and GDM-IR subgroups (at least 59 needed per group) and the population with NGT. Statistical analyses were performed using STATA software version 16.0 (StataCorp, USA). The significance threshold was set at *p*<0.05.

## Results

### Description of the cohort

The baseline characteristics of 1308 pregnant women are outlined in Table 1. The women were aged 31.5±5.0 years (mean ± SD) with a BMI of 33.0±6.8 kg/m^2^ at enrolment (28.2±2.2 weeks’ gestation). Women were predominantly multiparous (60.4%) and had self-reported ethnicity comparable with the UK population as a whole (82.5% white, 4.7% Asian, 9.7% black, 3.1% other ethnicity).

**Table 1:** Baseline characteristics of participants in the OPHELIA study (*n*=1308) categorised according to glucose tolerance and GDM pathophysiology

	*n*	All women (*n*=1308)	NGT (reference) (*n*=1027)	GDM-IR (*n*=135)	GDM-IS (*n*=73)	GDM-both (*n*=2)	GDM-neither (*n*=71)
Maternal age (years)	1308	31.5 ± 5.0	31.2 ± 4.9	31.7 ± 4.9	34.3 ± 4.8***	30.7 ± 0.8	32.1 ± 5.4
BMI at enrolment (kg/m^2)^	1249	33.0 ± 6.8	32.4 ± 6.5	38.2 ± 6.3***	30.1 ± 5.3**	44.8 ± 13.0	35.2 ± 7.0***
Gestational weight gain pre-enrolment (kg)	1243	7.2 ± 6.0	7.2 ± 5.5	7.9 ± 8.6	6.5 ± 5.1	3.5 ± 0.1	7.1 ± 8.3
Ethnicity^a^							
White		1079 (82.5)	836 (81.4)	117 (86.7)	62 (84.9)	0	64 (90.1)
Asian		61 (4.7)	53 (5.2)	3 (2.2)	2 (2.7)	2 (100.0)	1 (1.4)
Black		127 (9.7)	101 (9.8)	15 (11.1)	6 (8.2)	0	5 (7.0)
Other		41 (3.1)	37 (3.6)	0	3 (4.1)	0	1 (1.4)
Multiparous	1308	790 (60.4)	618/ (60.2)	73 (54.1)	49 (67.1)	1 (50.0)	49 (69.0)
Gestational age at OGTT	1289	28.2 ± 2.2	28.2 ± 2.0	28.1 ± 2.9	28.2 ± 2.4	27.9 ± 0.9	28.3 ± 2.8
HbA_1c_ (mmol/mol)	1236	31.7 ± 3.6	31.0 ± 3.1	34.7 ± 4.2***	33.5 ± 4.2***	39.0 ± 8.5	32.9 ± 3.8***
HbA_1c_ (%)	1236	5.05 ± 0.33	4.99 ± 0.29	5.32 ± 0.39	5.21 ± 0.39	5.72 ± 0.78	5.16 ± 0.35
Systolic BP (mmHg)	1030	117.1 ± 12.9	116.3 ± 12.8	122.6 ± 12.2***	117.9 ± 12.1	126.0 ± 8.5	118.5 ± 14.7
Diastolic BP (mmHg)	1030	66.5 ± 8.8	66.0 ± 8.8	70.5 ± 8.6***	65.3 ± 7.6	62.5 ± 10.6	68.0 ± 9.2
Insulin at 0 min (pmol/l)	1274	111.3 ± 85.9	99.2 ± 64.5	238.1 ± 141.0***	61.1 ± 22.4***	117.3 ± 3.2	91.4 ± 19.9
Insulin at 120 min (pmol/l)	1251	679.0 ± 570.2	580.4 ± 487.1	1342.3 ± 809.5***	593.6 ± 305.8	803.4 ± 336.1	863.0 ± 388.0***
Standard glucose processing							
OGTT 0 h glucose (mmol/l)	1300	4.4 ± 0.5	4.3 ± 0.3	5.0 ± 0.6***	4.7 ± 0.6***	6.6 ± 0.8	4.5 ± 0.4***
OGTT 2 h glucose (mmol/l)	1296	5.8 ± 1.4	5.4 ± 1.0	7.1 ± 1.5***	7.8 ± 1.7***	11.1 ± 1.3	7.5 ± 1.1***
Matsuda index	1226	6.2 ± 4.7	7.0 ± 4.9	2.0 ± 0.8***	6.1 ± 2.8	2.3 ± 0.7	3.9 ± 1.0***
HOMA2-S	1267	78.5 ± 57.5	84.4 ± 59.7	28.3 ± 9.6***	106.0 ± 51.9***	43.5 ± 0.1	64.2 ± 15.6**
HOMA2-B	1267	191.7 ± 85.1	188.9 ± 80.1	264.0 ± 108.3***	115.6 ± 22.3***	97.6 ± 25.3	170.9 ± 30.8
Enhanced glucose processing							
OGTT 0 h glucose (mmol/l)	1273	5.0 ± 0.5	4.9 ± 0.3	5.8 ± 0.6***	5.4 ± 0.8***	7.2 ± 0.8	5.1 ± 0.4***
OGTT 2 h glucose (mmol/l)	1250	6.4 ± 1.4	5.9 ± 1.0	7.8 ± 1.6***	8.3 ± 1.7***	9.4 ± 2.2	8.3 ± 1.0***
Matsuda index	1236	5.5 ± 4.2	6.2 ± 4.4	1.8 ± 0.7***	5.4 ± 2.5	2.2 ± 0.4	3.4 ± 0.9***
HOMA2-S	1272	75.7 ± 55.5	81.4 ± 57.8	27.3 ± 9.2***	101.6 ± 49.1***	42.7 ± 0.1	62.0 ± 15.1**
HOMA2-B	1272	147.7 ± 61.2	146.6 ± 59.3	198.3 ± 67.1***	87.4 ± 13.3***	83.4 ± 18.5	131.0 ± 20.0*
On no medication for GDM	1299	1168/1299 (89.9)	989/1019 (97.1)	95/135 (70.4)***	35/72 (47.9)***	1/2 (50.0)	48/71 (67.6)***
Outcomes							
Pre-eclampsia	1280	16/1280 (1.3)	9/1003 (0.9)	4/134 (3.0)*	1/71 (1.4)	0/2 (0.0)	2/70 (2.9)
Gestational age at birth (weeks)	1284	39.3 ± 1.7	39.4 ± 1.7	38.9 ± 1.7***	38.8 ± 1.5**	40.3 ± 1.6	38.9 ± 1.7*
Preterm delivery	1284	68/1284 (5.3)	47/1007 (4.7)	9/134 (6.7)	7/71 (9.9)	0/2 (0.0)	5/70 (7.1)
SVD	1308	666/1308 (50.9)	541/1027 (52.7)	57/135 (42.2)*	35/73 (47.9)	0/2 (0.0)	33/71 (46.5)
Caesarean delivery	1308	468/1308 (35.8)	353/1027 (34.4)	64/135 (47.4)**	23/73 (31.5)	1/2 (50.0)	27/71 (38.0)
Ventouse delivery	1308	41/1308 (3.1)	29/1027 (2.8)	2/135 (1.5)	5/73 (6.8)	1/2 (50.0)	4/71 (5.6)
Forceps delivery	1308	107/1308 (8.2)	82/1027 (8.0)	11/135 (8.2)	8/73 (11.0)	0/2 (0.0)	6/71 (8.5)
Sex of the infant (male)	1275	646/1275 (50.7)	508/999 (50.9)	66/133 (49.6)	35/71 (49.3)	1/2 (50.0)	36/70 (51.4)
Birthweight INTERGROWTH centile	1272	65.1 ± 27.2	63.9 ± 27.2	73.0 ± 26.4***	67.7 ± 26.0	61.2 ± 42.7)	63.9 ± 27.7
Birthweight GROW centile	1280	47.1 ± 29.6	45.5 ± 29.0	57.4 ± 32.3***	52.8 ± 29.5*	41.9 ± 32.9)	45.5 ± 30.2
LGA INTERGROWTH	1272	307/1272 (24.1)	220/996 (22.1)	51/133 (38.4)***	19/71 (26.8)	1/2 (50.0)	16/70 (22.9)
LGA GROW	1280	140/1280 (10.9)	87/1003 (8.7)	34/134 (25.4)***	10/71 (14.1)	0/2 (0.0)	9/70 (12.9)
PPH	1280	441/1280 (34.5)	330/1003 (32.9)	54/134 (40.3)	27/71 (38.0)	2/2 (100.0)	28/70 (40.0)
Neonatal hypoglycaemia	1280	28/1280 (2.2)	17/1003 (1.7)	5/134 (3.7)	3/71 (4.2)	0/2 (0.0)	3/70 (4.3)
Neonatal jaundice	1280	82/1280 (6.4)	55/1003 (5.5)	17/134 (12.7)***	5/71 (7.0)	0/2 (0.0)	5/70 (7.1)
NICU admission	1280	112/1280 (8.8)	84/1003 (8.4)	12/134 (9.0)	8/71 (11.3)	0/2 (0.0)	8/70 (11.4)

Categorical data are expressed as *n* (%); continuous data are expressed as means ± SD

Women with NGT were compared to women with insulin-resistant GDM (GDM-IR; HOMA2-S <25th percentile and HOMA2-B ≥25th percentile), insulin-insufficient GDM (GDM-IS; HOMA2-B <25th percentile and HOMA2-S ≥25th percentile), both insulin-resistant and insulin-insufficient GDM (GDM-both; HOMA2-B <25th percentile and HOMA2-S <25th percentile) and GDM with no evidence of insulin resistance or insulin insufficiency (GDM-neither; HOMA2-B ≥25th percentile and HOMA2-S ≥25th percentile)

Glucose concentrations were measured using the enhanced sampling process (*n*=1273); HOMA scores were available for 1272 participants as one insulin sample was unsuitable for analysis and therefore HOMA indices could not be calculated

Differences in sample size (*n*) across analyses reflect missing data arising from incomplete records or unavailable measurements for some participants

^a^*p*<0.001 across all groups

Testing of categorical data was performed using the χ^2^ test, except for ethnicity; continuous testing of continuous data was performed using unadjusted linear regression: *p<0.05; **p<0.01; ***p<0.001 when compared with the NGT group

NICU, neonatal intensive care unit; PPH, postpartum haemorrhage; SVD, spontaneous vaginal delivery

### Diagnostic capability: precision categorisation according to pathophysiology of GDM

**Fig. 1:**
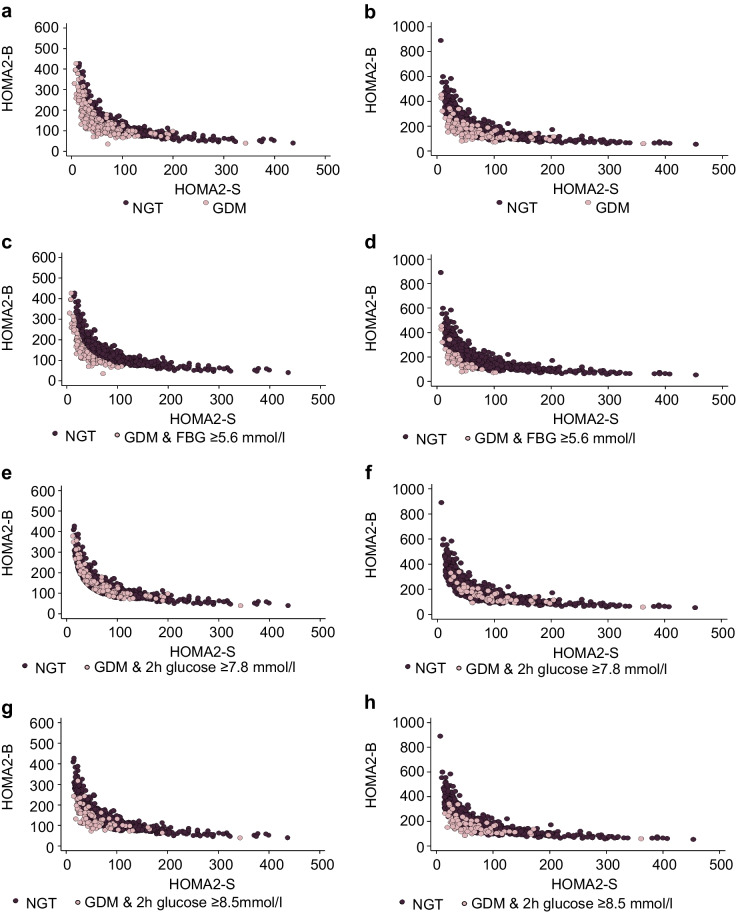
Comparison of HOMA2-S and HOMA2-B values categorised by whether women had NGT or GDM (**a**, **b**), according to the presence of fasting hyperglycaemia (**c**, **d**) or post-load hyperglycaemia (**e**, **f**) at the NICE threshold, and according to the presence of post-load hyperglycaemia (**g**, **h**) at the threshold set by the International Association of the Diabetes and Pregnancy Study Groups (IADPSG). Some women with GDM due to post-load hyperglycaemia have HOMA2-S and HOMA2-B values that are equivalent to those in women with NGT. Values were obtained using enhanced glucose processing (**a**, **c**, **e**, **g**) and standard glucose processing (**b**, **d**, **f**, **h**)

In women with NGT, the mean HOMA2-S (± SD) was 84.4±59.7 using standard processing and 81.4±57.8 using enhanced processing. The mean HOMA2-B (± SD) was 188.9±80.1 using standard processing and 146.6±59.3 using enhanced processing. As expected, most women with GDM had evidence of reduced HOMA2-B or HOMA2-S on enhanced or standard processing, but some women had HOMA2-B and HOMA2-S levels that overlapped with those of women with NGT (Fig. 1a, b). This overlap was more pronounced for post-load hyperglycaemia rather than fasting hyperglycaemia (Fig. 1c–f). This was not explained by the low threshold for diagnosis at the 2 h post-load timepoint when using the NICE guidelines, as it was also evident when a higher threshold of 8.5 mmol/l was used. This is the 2 h threshold according to the International Association of the Diabetes and Pregnancy Study Groups (IADPSG) criteria [19] (Fig. 1g, h).

**Fig. 2:**
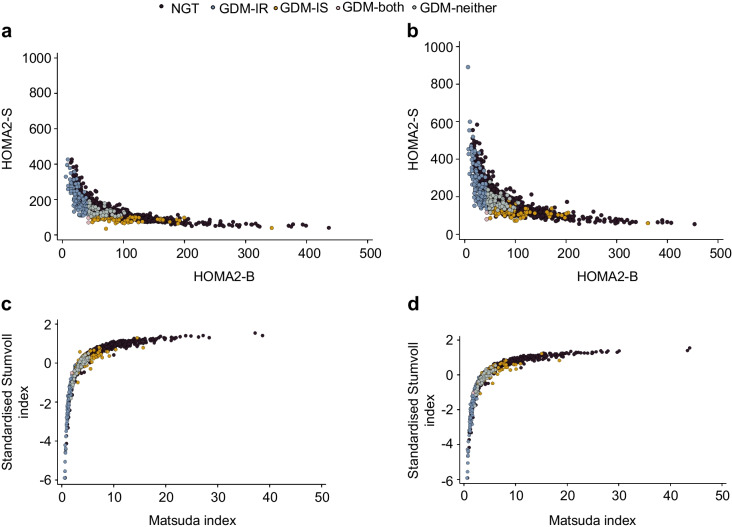
Comparison of HOMA2-S and HOMA2-B values (**a**, **b**) in women with NGT and women with GDM categorised by the presence of insulin resistance (HOMA2-S <25th percentile), insulin insufficiency (HOMA2-B <25th percentile), both or neither. Values were obtained using enhanced glucose processing (**a**) and standard glucose processing (**b**). Women with GDM but normal indices of insulin resistance or insulin secretion were also identified when alternative indices were used (**c**, **d**)

Using enhanced processing, the thresholds representing the 25th percentile for the population with NGT were 39.2 and 103.6 for HOMA2-S and HOMA2-B, respectively. Using these thresholds, 1027 of the 1308 women (78.5%) were categorised as having NGT, and 281 (21.5%) were categorised as having GDM. Of those classified as having GDM, 135 (48.0%) were categorised as having insulin-resistant GDM (GDM-IR), 73 (26.0%) were categorised as having insulin-insufficient GDM (GDM-IS), and two (0.7%) were categorised as having both insulin-resistant and insulin-insufficient GDM (GDM-both; ESM Fig. 1). However, the GDM in 71 (25.3%) of women was not explained by this classification, with these women having both HOMA2-S and HOMA2-B ≥ 25th centile (GDM-neither) (Fig. 2a, b).

This new subgroup of women (GDM-neither) had a higher BMI than women with NGT (35.2±7.0 vs 32.4±6.5 kg/m^2^; *p*<0.001) but were indistinguishable from other GDM subgroups based on clinical characteristics and medical history (Table 1). Compared with NGT, they had higher fasting glucose (5.1±0.4 vs 4.9±0.3 mmol/l; *p*<0.001; enhanced processing) and a similar fasting insulin (91.4±19.9 vs 99.2±64.5 pmol/l; *p*>0.05), suggesting that they were relatively insulin-sensitive in the fasting state (Table 1; Fig. 2a, b). However, they developed marked postprandial hyperglycaemia compared with the NGT group (mean 2 h glucose 8.3±1.0 vs 5.9±1.0 mmol/l; *p*<0.001; enhanced processing), with moderate hyperinsulinaemia (863.0±388.0 vs 580.4±487.1 pmol/l; *p*<0.001 suggesting an isolated defect in insulin sensitivity in the fed state. Overall, the women in this group had a modest reduction in insulin sensitivity and insulin secretion, with mean values less than one SD below the mean for the population with NGT (HOMA2-S: 62.0±15.1 vs 81.4±57.8 in NGT women, *p*=0.003; HOMA2-B: 131.0±20.0 vs 146.6±59.3 in NGT women, *p*=0.027; Table 1). As fasting glucose and insulin were normal, the specific defect in glucose handling in the post-load state was not adequately captured by the HOMA2-B or HOMA2-S indices (Fig. 1).

We assessed whether using different indices of insulin secretion and sensitivity would improve the categorisation and prevent a group of women being uncategorised. However, replacing HOMA2-B and HOMA2-S with the Stumvoll and Matsuda indices also identified women who had comparable insulin resistance and insulin secretion to the NGT population (Fig. 2c, d). Increasing the HOMA2-S and HOMA2-B thresholds of normality to ≥ 50^th^ centile did not fully resolve this issue either.

### Prognostic capability: clinical relevance of precision categorisation of GDM

As expected, women with GDM gave birth at a slightly earlier gestational age compared to women with NGT (Table 1, ESM Table 2). Very few women had evidence of both insulin-resistant and insulin-insufficient GDM in this cohort, preventing examination of their pregnancy outcomes.

Compared to women with NGT, women with insulin-resistant GDM had higher BMI (38.2±6.3 vs 32.4±6.5 kg/m^2^), systolic BP (122.6±12.2 vs 116.3±12.8 mmHg) and diastolic BP (70.5±8.6 vs 66.0±8.8 mmHg), and had a higher rate of pre-eclampsia (3.0% vs 0.9%, *p*=0.033), Caesarean delivery (47.4% vs 34.4%, *p*=0.003) and LGA (INTERGROWTH; 38.4% vs 22.1%, *p*<0.001). Offspring birthweight was higher (mean 9.1 centiles higher for INTERGROWTH; 11.9 centiles higher for GROW, *p*<0.001 for each) and more infants developed jaundice (12.7% vs 5.5%, *p*=0.001).

Compared to women with NGT, women with insulin-insufficient GDM were older (34.3±4.8 vs 31.2±4.9 years; *p*<0.001) and leaner (30.1±5.3 vs 32.4±6.5 kg/m^2^; *p*=0.006), and their offspring were approximately seven centiles higher in terms of birthweight (GROW, *p*=0.045; not significant for INTERGROWTH). Women categorised as GDM-neither, who had GDM with HOMA2-B and HOMA2-S above the 25th centile, had a higher BMI (35.2±7.0 vs 32.4±6.5 kg/m^2^; *p*<0.001) compared to those with NGT. Women with GDM-neither had comparable pregnancy outcomes to women with NGT.

While differences were evident when comparing the individual subgroups to women with NGT, there were very few significant differences in pregnancy outcomes between groups (ESM Table 2). Women with insulin-resistant GDM had higher rates of Caesarean delivery (47.4 vs 31.5%, *p*=0.026) and lower rates of ventouse delivery (1.5 vs 6.8%, *p*=0.040) compared to women with insulin-insufficient GDM. Women with GDM-neither had babies of comparable birthweight to those of the population with NGT, but lower than those of women with insulin-resistant GDM.

The associations between HOMA2-S and HOMA2-B, maternal BMI and fasting glucose with pregnancy outcomes are shown in Table 2. Each marker was associated with at least one pregnancy outcome, and all markers were associated with LGA (INTERGROWTH); this remained significant after adjustment for maternal age and GDM diagnosis. However, no marker showed strong predictive capability using AUROC or AUPRC (Table 3). BMI predicted a similar number of outcomes to HOMA2-S, suggesting that there was no additional predictive value in adding HOMA2-S. BMI was the strongest predictor of admission to the neonatal intensive care unit. We evaluated the additive effect of fasting glucose and HOMA scores (ESM Table 3), but they showed similar results to a model using standard maternal characteristics.

**Table 2:** Associations of HOMA2-S and HOMA2-B, maternal BMI and fasting glucose with pregnancy outcomes

	*n*	HOMA2-S	HOMA2-B	BMI	Fasting glucose
Unadjusted outcomes					
Pre-eclampsia	1245	0.25 (0.08, 0.74)*	1.61 (1.11, 2.32)*	1.66 (1.26, 2.19)***	1.43 (1.05, 1.95)*
Preterm delivery	1249	0.96 (0.85, 1.09)	0.96 (0.77, 1.21)	0.83 (0.68, 1.01)	1.09 (0.90, 1.31)
SVD	1272	1.13 (0.99, 1.30)	0.99 (0.79, 1.23)	0.86 (0.73, 1.03)	0.89 (0.82, 0.96)**
Caesarean delivery	1272	0.86 (0.76, 0.97)*	1.06 (0.85, 1.32)	1.32 (1.15, 1.52)***	1.10 (1.04, 1.23)*
Ventouse delivery	1272	1.22 (0.98, 1.53)	0.53 (0.34, 0.82)**	0.72 (0.52, 1.00)	1.10 (0.87, 1.38)
Forceps delivery	1272	0.89 (0.71, 1.10)	1.06 (0.78, 1.45)	0.84 (0.70, 0.99)*	1.11 (0.96, 1.27)
LGA INTERGROWTH	1237	0.77 (0.69, 0.85)***	1.17 (1.04, 1.32)**	1.46 (1.23, 1.74)***	1.24 (1.10, 1.41)*
LGA GROW	1245	0.70 (0.58, 0.82)***	1.17 (1.02, 1.34)*	1.24 (1.08, 1.43)**	1.33 (1.14, 1.56)***
PPH	1245	0.82 (0.69, 0.98)*	1.08 (0.81, 1.43)	1.16 (1.04, 1.29)**	1.12 (1.00, 1.25)*
Neonatal hypoglycaemia	1245	0.63 (0.26, 1.51)	1.19 (0.77, 1.85)	1.28 (0.80, 2.04)	1.23 (0.86, 1.77)
Neonatal jaundice	1245	0.88 (0.69, 1.14)	1.03 (0.80, 1.32)	0.88 (0.74, 1.05)	1.04 (0.85, 1.30)
NICU admission	1245	0.78 (0.71, 0.86)***	1.14 (1.06, 1.24)**	1.34 (1.10, 1.64)**	1.03 (0.92, 1.15)
Adjusted outcomes					
Pre-eclampsia	1245	0.28 (0.11, 0.73)**	1.55 (1.06, 2.27)*	1.60 (1.18, 2.18)*	1.39 (1.09, 1.77)*
Preterm delivery	1249	0.97 (0.83, 1.13)	0.99 (0.77, 1.27)	0.82 (0.66, 1.01)	1.01 (0.81, 1.25)
SVD	1272	1.15 (1.01, 1.30)*	0.97 (0.77, 1.21)	0.86 (0.72, 1.01)	0.89 (0.82, 0.97)*
Caesarean delivery	1272	0.84 (0.76, 0.94)*	1.09 (0.87, 1.37)	1.34 (1.17, 1.54)***	1.08 (1.01, 1.16)*
Ventouse delivery	1272	1.25 (1.01, 1.54)*	0.51 (0.34, 0.77)**	0.71 (0.50, 1.01)	1.07 (0.77, 1.47)
Forceps delivery	1272	0.89 (0.71, 1.11)	1.06 (0.76, 1.46)	0.83 (0.70, 0.99)*	1.13 (0.95, 1.34)
LGA INTERGROWTH	1237	0.76 (0.68, 0.85)***	1.17 (1.04, 1.32)*	1.47 (1.24, 1.74)***	1.30 (1.15, 1.47)***
LGA GROW	1245	0.70 (0.59, 0.83)***	1.16 (1.00, 1.36)	1.23 (1.09, 1.40)**	1.37 (1.18, 1.60)***
PPH	1245	0.82 (0.69, 0.97)*	1.11 (0.83, 1.47)	1.17 (1.04, 1.31)*	1.08 (0.96, 1.21)
Neonatal hypoglycaemia	1245	0.68 (0.32, 1.48)	1.21 (0.77, 1.92)	1.26 (0.83, 1.92)	1.03 (0.82, 1.29)
Neonatal jaundice	1245	0.93 (0.60, 1.44)	0.96 (0.74, 1.25)	0.84 (0.71, 1.00)*	0.99 (0.79, 1.24)
NICU admission	1245	0.78 (0.71, 0.85)***	1.13 (1.05, 1.22)**	1.36 (1.11, 1.65)*	1.01 (0.91, 1.12)

Values are ORs (95% CI) for a one SD increase in each measure

Glucose concentrations were measured using the enhanced sampling process (*n*=1273). HOMA scores were available for 1272 participants, as one insulin sample was unsuitable for analysis therefore HOMA indices could not be calculated

The lower section shows the adjusted analysis: all results were adjusted for GDM diagnosis (by NICE criteria) and maternal age; neonatal outcomes (postpartum haemorrhage, neonatal hypoglycaemia, neonatal jaundice and NICU admission) were also adjusted for gestational age at delivery. Neonatal hypoglycaemia is reported for completeness, but these results are subject to ascertainment bias, as infants of mothers without GDM are not routinely tested. The model used was a multilevel model with study centre as the cluster for both adjusted and unadjusted analyses

Unadjusted logistic regression was used to determine the significance values for the outcomes in the upper section, whilst logistic regression adjusted for GDM diagnosis and maternal age was used to calculate the significance values for outcomes in the lower section. The significance level is indicated by asterisks: *p<0.05; **p<0.01; ***p<0.001

Differences in sample size (*n*) across analyses reflect missing data arising from incomplete records or unavailable measurements for some participants. Only participants with complete data for HOMA indices, maternal BMI and fasting glucose, and each pregnancy outcome were included in the analyses, resulting in a lower sample size than the total cohort

NICU, neonatal intensive care unit; PPH, postpartum haemorrhage; SVD, spontaneous vaginal delivery

**Table 3:** The predictive capability of HOMA2-S, HOMA2-B, maternal BMI and fasting glucose (mmol/l) for pregnancy outcomes across the total cohort (*n*=1273)

Outcome	HOMA2-S AUROC	HOMA2-S AUPRC	BMI AUROC	BMI AUPRC	HOMA2-B AUROC	HOMA2-B AUPRC	Fasting glucose AUROC	Fasting glucose AUPRC	*p* value for HOMA2-S vs BMI (AUROC)	*p* value for all predictors (AUROC)
Pre-eclampsia	0.30 (0.15, 0.45)	0.01 (0.01)	0.69 (0.54, 0.83)	0.02 (0.01)	0.69 (0.53, 0.84)	0.04 (0.01)	0.54 (0.36, 0.71)	0.03 (0.01)	0.854	0.073
Preterm delivery	0.49 (0.42, 0.57)	0.05 (0.05)	0.46 (0.39, 0.53)	0.05 (0.05)	0.50 (0.42, 0.57)	0.05 (0.05)	0.53 (0.45, 0.61)	0.07 (0.05)	0.129	0.395
SVD	0.52 (0.49, 0.55)	0.54 (0.51)	0.45 (0.42, 0.48)	0.48 (0.51)	0.49 (0.46, 0.52)	0.51 (0.51)	0.47 (0.43, 0.50)	0.48 (0.51)	0.132	0.129
Caesarean delivery	0.54 (0.51, 0.58)	0.33 (0.36)	0.59 (0.56 ,0.63)	0.42 (0.35)	0.54 (0.51, 0.57)	0.37 (0.36)	0.53 (0.49, 0.56)	0.39 (0.36)	0.003**	0.0006***
Ventouse delivery	0.39 (0.31, 0.47)	0.04 (0.03)	0.39 (0.30, 0.48)	0.03 (0.03)	0.35 (0.27, 0.44)	0.02 (0.03)	0.51 (0.42, 0.61)	0.04 (0.03)	0.960	0.080
Forceps delivery	0.52 (0.46, 0.58)	0.08 (0.08)	0.45 (0.39, 0.51)	0.08 (0.09)	0.50 (0.44, 0.56)	0.08 (0.08)	0.54 (0.49, 0.60)	0.09 (0.08)	0.015*	0.053
LGA INTERGROWTH	0.58 (0.54, 0.62)	0.21 (0.24)	0.61 (0.57, 0.64)	0.32 (0.24)	0.56 (0.52, 0.59)	0.27 (0.24)	0.58 (0.54, 0.62)	0.29 (0.24)	0.129	0.0002***
LGA GROW	0.60 (0.54, 0.65)	0.09 (0.11)	0.57 (0.52, 0.62)	0.13 (0.11)	0.56 (0.51, 0.61)	0.13 (0.11)	0.60 (0.54, 0.65)	0.17 (0.11)	0.276	0.014*
PPH	0.54 (0.51, 0.57)	0.31 (0.35)	0.55 (0.51, 0.58)	0.38 (0.35)	0.53 (0.50, 0.57)	0.36 (0.35)	0.53 (0.50, 0.57)	0.38 (0.35)	0.693	0.393
Neonatal hypoglycaemia	0.59 (0.49, 0.69)	0.02 (0.02)	0.56 (0.46, 0.67)	0.03 (0.02)	0.56 (0.45, 0.67)	0.03 (0.02)	0.57 (0.45, 0.69)	0.03 (0.02)	0.553	0.455
Neonatal jaundice	0.54 (0.47, 0.60)	0.06 (0.06)	0.47 (0.40, 0.53)	0.06 (0.07)	0.52 (0.46, 0.58)	0.07 (0.06)	0.54 (0.47, 0.60)	0.07 (0.06)	0.014*	0.093
NICU admission	0.56 (0.55, 0.85)	0.08 (0.09)	0.69 (0.54, 0.83)	0.12 (0.09)	0.56 (0.51, 0.62)	0.10 (0.09)	0.51 (0.44, 0.57)	0.09 (0.09)	0.848	0.083

Data are AUROC (with 95% CI) and AUPRC (with percentage of positive cases for each outcome)

Differences in sample size (*n*) across analyses reflect missing data arising from incomplete records or unavailable measurements for some participants. Only participants with complete data for HOMA indices, maternal BMI and fasting glucose, and each pregnancy outcome were included in the analyses, resulting in a lower sample size than the total cohort

Differences between the AUROC values, determined using the χ^2^ test, are indicated by *p* values in the final two columns of the table for the comparison with HOMA2-S and maternal BMI and for the comparison amongst all predictors: *p<0.05; **p<0.01; ***p<0.001

NICU, neonatal intensive care unit; PPH, postpartum haemorrhage; SVD, spontaneous vaginal delivery

### Robustness of categorisation based on specimen handling method

Standard specimen handling resulted in fasting and post-load glucose levels that were approximately 0.6 mmol/l lower than glucose concentrations measured using enhanced specimen processing procedures (Table 1). However, when the indices were recalculated based on glucose values obtained using standard processing, the values for the 25th centile of HOMA2-B and HOMA2-S in the NGT population were slightly different (HOMA2-B: 136.6% vs 178.3%; HOMA2-S: 66.2% vs 65.1%; enhanced processing vs standard processing). Despite these differences, precision categorisation yielded similar results despite the lower numbers of women with GDM overall and the lower numbers in each subgroup. The proportion of patients allocated to each subgroup was similar (ESM Fig. 1).

## Discussion

This study identified that the HOMA2-B/HOMA2-S precision categorisation of GDM was robust to changes in specimen processing, but that these indices provided little additional diagnostic or prognostic information beyond that which is already available using existing antenatal monitoring. However, we identified a novel subgroup (accounting for 25% of patients with GDM) with an isolated postprandial defect in glucose handling and normal pregnancy outcomes; these women had comparable values of HOMA2-S and HOMA2-B to those for normoglycaemic pregnant women.

Although HOMA2-B/HOMA2-S precision categorisation of GDM makes sense from a pathophysiological perspective, in practice neither HOMA2-S nor HOMA2-B are strong predictors of GDM or pregnancy outcomes (Tables 2 and 3). We found that, despite being an imperfect predictor, maternal BMI shows equivalent or better predictive capability over pregnancy outcomes compared with HOMA scores, and is routinely measured in clinical practice without the need for insulin determination. Our data suggest that BMI may be a more successful and equitable precision marker than mathematical measures of insulin secretion or resistance. This is unsurprising as BMI in normoglycaemic pregnant women is associated with increased rates of operative delivery [20], babies who are large for gestational age [21, 22], hypertensive disorders [22, 23] and preterm birth [24]. In women with diabetes, BMI also correlates with the onset and severity of hyperglycaemia [25, 26] and the severity of insulin resistance [27], which are additive risk factors for suboptimal pregnancy outcomes [28]. BMI offers practical advantages as a prognostic marker, being widely used, easily measured and cost-effective, with minimal resources and training needed.

### Strengths and weaknesses

We recruited an antenatal population of women with one or more risk factors for GDM, who had specimens taken using standard and enhanced protocols at 0 and 120 min during a 75 g antenatal OGTT. We calculated multiple indices for insulin resistance and insulin secretory function but were limited to indices that use these timepoints. Additional sampling at 60 min may have improved the performance of indices of insulin secretion and allowed comparison with GDM diagnosed using other diagnostic criteria, as there is mounting evidence for the value of the 60 min test [29, 30]. Our standard specimen processing procedure was designed to replicate clinical care and local sample pre-analytical and analytical procedures at each of our nine sites. While this introduced variation, all laboratories used in this study were accredited under the UK Accreditation Service, and can demonstrate acceptable analytical performance during the study period. Citrate tubes were not used for glucose assessment as these were not widely available at the time of study initiation [31].

Only 281 of the 1308 women had GDM, although all women in our population had at least one risk factor for GDM, which may have influenced glucose, insulin, HOMA2-S and HOMA2-B values for the NGT population. This is important as it reflects the thresholds used (< 25th centile of normal population) for HOMA2-S and HOMA2-B, and thus may have changed the number of women allocated to the insulin-resistant and insulin-insufficient subgroups. We considered that it was more robust to use the enhanced glucose measurements for calculation of HOMA scores and categorisation into groups. However, clinical diagnosis was based on standard processing of venous glucose levels, causing heterogeneity within our group, as not all women had access to treatment. Treatment itself may reduce the predictive capability of any measure obtained at 28 weeks, but this cannot be avoided while conducting an ethical study.

### Relevance to other studies

Previous work by Powe et al used HOMA2-S and HOMA2-B thresholds of the 25th centile to categorise women with GDM into insulin-resistant and insulin-insufficient subgroups, or both [4]. In their population in the USA, the proportions of women with insulin-resistant GDM (51%), insulin-insufficient GDM (30%) were similar to those for our cohort; however, the proportion of women categorised as having GDM with both insulin resistance and insulin insufficiency differed (18% GDM-mixed [the category referred to as ‘GDM-both’ in our study]), and only one woman was unclassified, compared with 71 (25.3%) in our study [4]. In contrast to the study by Powe et al and our work, Benhalima et al found a greater proportion of women with insulin-resistant GDM (83%), defined as having a Matsuda index below the 50th percentile of that for women with NGT [5]. A study by Liu et al in China found that 17% of women with GDM could be categorised as insulin-resistant, 21% were categorised as insulin-insufficient and 38% were categorised as mixed. Liu et al used thresholds of the 25th percentile of the Matsuda index and oral disposition index to distinguish insulin sensitivity and insulin secretion [32]. Similarly to our study, Liu et al also identified a group of women with GDM and normal indices (representing 23.8% of women with GDM), but these participants were not further characterised. While use of HOMA2-S and HOMA2-B can identify women with GDM and near-normal indices, suggesting valid pathophysiological classification, they probably miss isolated postprandial insulin sensitivity defects, indicating imperfect detection of more subtle abnormalities. Differences in approaches to screening for hyperglycaemia in pregnancy, diagnostic criteria for GDM, the definition of NGT and the population prevalence of obesity may influence the thresholds for insulin resistance and insulin insufficiency, creating differences between the performance of this precision approach in different populations.

Use of the HOMA scores is undoubtedly convenient, but using improved measures of insulin resistance or insulin secretion may improve the performance of this precision approach. Cohen et al attempted to validate the HOMA-IR score longitudinally in six women who were assessed using euglycaemic–hyperinsulinaemic clamps in pregnancy and postnatally [11]. They identified large inter-individual differences in the relationship between HOMA-IR and glucose disappearance on the clamp, and concluded that the HOMA-IR score lacked sensitivity for individual use (*R*^2^ = 0.45) [11]. Kirwan et al found the Matsuda index to be most closely associated with clamp-derived insulin resistance estimates in pregnancy, with HOMA2-S showing the weakest associations (*R*^2^ = 0.52–0.61) [10]. However, we found similar results when using a Matsuda–Stumvoll classification instead of HOMA2-S and HOMA2-B. Despite these limitations, HOMA scores are widely used in the pregnancy literature, for example as primary outcomes in a randomised controlled trial [33] and to assess change in response to lifestyle interventions [34], and are associated with relevant pregnancy outcomes, including the development of GDM [35, 36].

Although the optimal criteria for precision categorisation of women with GDM are still unclear, there is a growing body of evidence suggesting that insulin-resistant GDM is more strongly associated with suboptimal pregnancy outcomes, including pre-eclampsia, preterm delivery, LGA babies and neonatal hypoglycaemia, independently of maternal blood glucose levels or obesity [5, 32]. In the study by Benhalima et al, women with high levels of insulin resistance had higher rates of preterm delivery and neonatal hypoglycaemia, despite adjustment for confounding factors such as BMI and gestational weight gain [5]. Liu et al found that women with GDM with both insulin resistance and insulin insufficiency had a higher risk of LGA babies, but there was no other difference between the groups [32]. In this study, we also found that insulin-resistant GDM was associated with LGA babies. However, BMI offered similar prognostic information to the HOMA2-S score, and is more accessible in clinical practice internationally. Women with insulin-insufficient GDM appear to have pregnancy outcomes that are comparable to those of the normoglycaemic population [32], but women with altered insulin secretion still appear to have substantial risks of type 2 diabetes postpartum [29, 30].

### Main implications for future work

With rising population levels of obesity in many countries internationally, there is an urgent need to improve risk prediction in women with GDM, allowing time and resources to be targeted to the highest-risk pregnancies and to type 2 diabetes prevention [2, 3]. While novel precision approaches may help, widely available measurements such as BMI and glucose concentrations during an OGTT already provide rich prognostic information and could be used more effectively, as highlighted by a recent publication from the joint ADA/EASD Precision Medicine in Diabetes Initiative [2]. A binary diagnosis for GDM with a single treatment pathway overlooks the severity of hyperglycaemia and obesity, which are key determinants of outcomes in GDM.

A key question remains: what might we reasonably aim to achieve with a precision approach? The recent consensus report from Tobias et al as part of the ADA/EASD Precision Medicine in Diabetes Initiative highlights GDM prevention, diagnosis, treatment and prognosis as key areas where precision medicine can improve care [1]. Our study found that women with insulin-resistant GDM had higher rates of Caesarean delivery and gave birth to babies of higher birthweight. However, LGA babies can already be predicted using ultrasound measurements, and delivery modality is more likely to be affected by maternal BMI than maternal insulin resistance.

### Conclusion

Use of a precision medicine approach comprising HOMA2-S and HOMA2-B did not add useful diagnostic or prognostic information in a population of pregnant women at risk of GDM. However, it did identify a novel subgroup of GDM women with an isolated postprandial defect in glucose handling, which was not associated with substantial impairments in HOMA2-S or HOMA2-B. Further work is needed to identify a successful precision medicine approach to GDM.

## Supplementary Information

Below is the link to the electronic supplementary material.

ESM (PDF 219 KB)

## Data Availability

Data are available from the corresponding author upon request, subject to approval from the sponsor and study steering committee.

## Abbreviations

AUPRC: Area under the precision-recall curve
AUROC: Area under the receiver operating characteristic curve
GDM: Gestational diabetes mellitus
GDM-IR: Insulin-resistant GDM
GDM-IS: Insulin-insufficient GDM
GDM-both: Insulin-resistant and insulin-insufficient GDM
GDM-neither: GDM with no evidence of insulin resistance or insulin insufficiency
LGA: Large for gestational age
NGT: Normal glucose tolerance
