# Supplementary material for: A critique of measurement of defective insulin secretion and insulin sensitivity as a precision approach to gestational diabetes
Source: Diabetologia. 2024 Dec 2;68(4):752–65. doi: 10.1007/s00125-024-06334-x (PMC11950144; doi:10.1007/s00125-024-06334-x)

Title: A critique of measurement defective insulin secretion and insulin sensitivity as a precision approach to gestational diabetes.

Jones et al.

Electronic Supplementary Material

ESM Table 1. Protocols used to calculate indices of insulin sensitivity and secretion in this manuscript.

Method	Formula/calculator
**Matsuda**	$10{,}000/\sqrt{(G_0 \times I_0) \times (G_{120} \times I_{120})}$
**HOMA2-S**	https://www.dtu.ox.ac.uk/homacalculator/
**HOMA2-IR**	https://www.dtu.ox.ac.uk/homacalculator/
**Disposition**	$(I_{0-120})/(G_{0-120}) \times 1/I_0$
**Stumvoll**	$0.156 - 0.0000459 \times I_{120}$ (pmol/L) – $0.000321 \times I_0$ (pmol/L) – $0.00541 \times G_{120}$ (mmol/L)
**HOMA2-B**	https://www.dtu.ox.ac.uk/homacalculator/

$\mathbf{G_0}$ = fasting glucose (mmol/L), $\mathbf{G_{120}}$ = post load glucose (mmol/L), $\mathbf{I_0}$ = fasting insulin (pmol/L), $\mathbf{I_{120}}$ = post load insulin (pmol/L), **0.18** = conversion factor to transform blood glucose concentration from mmol/L into mg/ml, **6** = conversion factor to transform insulin concentration from pmol/L to µIU/mL, **VD** = volume distribution (150 x Body Weight (kg)).

ESM Table 2: Baseline characteristics of participants in the OPHELIA study with more detail of indices and treatments. Categorical testing using Chi squared. Continuous testing using unadjusted linear regression. Abbreviations; SVD, spontaneous vaginal delivery; LGA, Large for gestational age; PPH, post-partum haemorrhage; NICU, Neonatal intensive care unit.

	n	ALL WOMEN n=1308	0: NGT n=1027	1: GDM-IR n=135	2: GDM-IS n=73	3: GDM-both n=2	4: GDM-neither n=71	p 0 vs 1	p 0 vs 2	p 0 vs 4	p 1 vs 2	p 1 vs 4	p 2 vs 4
**Maternal age years**	1308	31.5 (5.0)	31.2 (4.9)	31.7 (4.9)	34.3 (4.8)	30.7 (0.8)	32.1 (5.4)	0.298	<0.001	0.157	<0.001	0.602	0.010
**BMI at enrolment**	1249	33.0 (6.8)	32.4 (6.5)	38.2 (6.3)	30.1 (5.3)	44.8 (13.0)	35.2 (7.0)	<0.001	0.006	<0.001	<0.001	0.002	<0.001
**Gestational weight gain pre-enrolment**	1243	7.2 (6.0)	7.2 (5.5)	7.9 (8.6)	6.5 (5.1)	3.5 (0.1)	7.1 (8.3)	0.199	0.376	0.936	0.226	0.494	0.608
**Ethnicity %**	White	1079/1308 (82.5%)	836/1027 (81.4%)	117/135 (86.7%)	62/73 (84.9%)	0	64/71 (90.1%)						
	Asian	61/1308 (4.7%)	53/1027 (5.2%)	3/135 (2.2%)	2/73 (2.7%)	2/2 (100.0%)	1/71 (1.4%)				p<0.001 across groups		
	Black	127/1308 (9.7%)	101/1027 (9.8%)	15/135 (11.1%)	6/73 (8.2%)	0	5/71 (7.0%)						
	Other	41/1308 (3.1%)	37/1027 (3.6%)	0	3/73 (4.1%)	0	1/71 (1.4%)						
**Multiparous**	1308	790/1308 (60.4%)	618/1027 (60.2%)	73/135 (54.1%)	49/73 (67.1%)	1/2 (50.0%)	49/71 (69.0%)	0.175	0.240	0.140	0.068	0.038	0.808
**Gestational age at OGTT**	1289	28.2 (2.2)	28.2 (2.0)	28.1 (2.9)	28.2 (2.4)	27.9 (0.9)	28.3 (2.8)	0.925	0.813	0.663	0.836	0.733	0.902
**HbA1c mmol/mol**	1236	31.7 (3.6)	31.0 (3.1)	34.7 (4.2)	33.5 (4.2)	39.0 (8.5)	32.9 (3.8)	<0.001	<0.001	<0.001	0.057	0.004	0.387
**HbA1c %**	1236	5.1 (2.5)	5.0 (2.4)	5.3 (2.5)	5.2 (2.5)	5.7 (2.9)	5.2 (2.5)	<0.001	<0.001	<0.001	0.057	0.004	0.387
**Systolic blood pressure mmHg**	1030	117.1 (12.9)	116.3 (12.8)	122.6 (12.2)	117.9 (12.1)	126.0 (8.5)	118.5 (14.7)	<0.001	0.351	0.217	0.032	0.062	0.824
**Diastolic blood pressure mmHg**	1030	66.5 (8.8)	66.0 (8.8)	70.5 (8.6)	65.3 (7.6)	62.5 (10.6)	68.0 (9.2)	<0.001	0.564	0.098	<0.001	0.094	0.095
**Insulin 0 min pmol/l**	1274	111.3 (85.9)	99.2 (64.5)	238.1 (141.0)	61.1 (22.4)	117.3 (3.2)	91.4 (19.9)	<0.001	<0.001	0.389	<0.001	<0.001	<0.001
**Insulin 120 pmol/l**	1251	679.0 (570.2)	580.4 (487.1)	1,342.3 (809.5)	593.6 (305.8)	803.4 (336.1)	863.0 (388.0)	<0.001	0.836	<0.001	<0.001	<0.001	<0.001
**STANDARD GLUCOSE PROCESSING**													
**OGTT 0 hr glucose mmol/l**	1300	4.4 (0.5)	4.3 (0.3)	5.0 (0.6)	4.7 (0.6)	6.6 (0.8)	4.5 (0.4)	<0.001	<0.001	<0.001	<0.001	<0.001	0.011
**OGTT 2 hr glucose mmol/l**	1296	5.8 (1.4)	5.4 (1.0)	7.1 (1.5)	7.8 (1.7)	11.1 (1.3)	7.5 (1.1)	<0.001	<0.001	<0.001	0.003	0.052	0.371
**Matsuda index**	1226	6.2 (4.7)	7.0 (4.9)	2.0 (0.8)	6.1 (2.8)	2.3 (0.7)	3.9 (1.0)	<0.001	0.096	<0.001	<0.001	<0.001	<0.001
**HOMA2-S**	1267	78.5 (57.5)	84.4 (59.7)	28.3 (9.6)	106.0 (51.9)	43.5 (0.1)	64.2 (15.6)	<0.001	0.001	0.003	<0.001	<0.001	<0.001
**HOMA2-IR**	1267	2.0 (1.4)	1.7 (1.1)	4.2 (2.2)	1.1 (0.4)	2.3 (0.0)	1.6 (0.4)	<0.001	<0.001	0.504	<0.001	<0.001	<0.001
**Disposition index**	1197	2,420.0 (3,033.2)	2,743.4 (3,182.7)	1,481.2 (2,822.3)	1,280.1 (1,334.1)	322.9 (34.4)	1,024.6 (520.6)	<0.001	<0.001	<0.001	0.526	0.143	0.138
**Stumvoll index**	1230	0.1 (0.0)	0.1 (0.0)	0.0 (0.1)	0.1 (0.0)	0.0 (0.0)	0.0 (0.0)	<0.001	0.820	<0.001	<0.001	<0.001	<0.001

HOMA2-B	1267	191.7 (85.1)	188.9 (80.1)	264.0 (108.3)	115.6 (22.3)	97.6 (25.3)	170.9 (30.8)	<0.001	<0.001	0.065	<0.001	<0.001	<0.001
**Continued**	**n**	**ALL WOMEN n=1308**	**0: NGT n=1027**	**1: GDM-IR n=135**	**2: GDM-IS n=73**	**3: GDM-both n=2**	**4: GDM-neither n=71**	**p 0 vs 1**	**p 0 vs 2**	**p 0 vs 4**	**p 1 vs 2**	**p 1 vs 4**	**p 2 vs 4**
**ENHANCED GLUCOSE PROCESSING**													
**OGTT 0 hr glucose mmol/l**	1273	5.0 (0.5)	4.9 (0.3)	5.8 (0.6)	5.4 (0.8)	7.2 (0.8)	5.1 (0.4)	<0.001	<0.001	<0.001	<0.001	<0.001	0.003
**OGTT 2 hr glucose mmol/l**	1250	6.4 (1.4)	5.9 (1.0)	7.8 (1.6)	8.3 (1.7)	9.4 (2.2)	8.3 (1.0)	<0.001	<0.001	<0.001	0.021	0.016	0.923
**Matsuda index**	1236	5.5 (4.2)	6.2 (4.4)	1.8 (0.7)	5.4 (2.5)	2.2 (0.4)	3.4 (0.9)	<0.001	0.095	<0.001	<0.001	<0.001	<0.001
**HOMA2-S**	1272	75.7 (55.5)	81.4 (57.8)	27.3 (9.2)	101.6 (49.1)	42.7 (0.1)	62.0 (15.1)	<0.001	0.002	0.003	<0.001	<0.001	<0.001
**HOMA2-IR**	1272	2.0 (1.5)	1.8 (1.1)	4.3 (2.3)	1.2 (0.4)	2.3 (0.0)	1.7 (0.4)	<0.001	<0.001	0.506	<0.001	<0.001	<0.001
**Disposition index**	1208	2,321.8 (3,259.5)	2,621.3 (3,435.4)	1,266.3 (2,301.6)	1,543.9 (2,472.8)	9,099.0 (12,499.8)	896.5 (688.9)	<0.001	0.006	<0.001	0.392	0.257	0.069
**Stumvoll index**	1236	0.1 (0.0)	0.1 (0.0)	0.0 (0.1)	0.1 (0.0)	0.0 (0.0)	0.0 (0.0)	<0.001	0.860	<0.001	<0.001	<0.001	<0.001
**HOMA2-B**	1272	147.7 (61.2)	146.6 (59.3)	198.3 (67.1)	87.4 (13.3)	83.4 (18.5)	131.0 (20.0)	<0.001	<0.001	0.027	<0.001	<0.001	<0.001
**MEDICATION FOR GDM**													
**On no medication for GDM**	1299	1168/1299 (89.9%)	989/1019 (97.1%)	95/135 (70.4%)	35/72 (47.9%)	1/2 (50.0%)	48/71 (67.6%)	<0.001	<0.001	<0.001	0.002	0.682	0.021
**Taking metformin**	1299	45/1299 (3.5%)	13/1019 (1.3%)	18/135 (13.3%)	10/72 (13.9%)	0/2 (0.0%)	4/71 (5.6%)	<0.001	<0.001	0.004	0.911	0.089	0.097
**Taking Novorapid**	1299	11/1299 (0.9%)	3/1019 (0.3%)	5/135 (3.7%)	1/72 (1.4%)	0/2 (0.0%)	2/71 (2.8%)	<0.001	0.138	0.002	0.344	0.738	0.551
**Taking Insulatard**	1299	17/1019 (1.3%)	2/1027 (0.2%)	8/135 (5.9%)	4/72 (5.6%)	1/2 (50.0%)	2/71 (2.8%)	<0.001	<0.001	<0.001	0.914	0.324	0.414
**Taking other insulin combinations**	1299	15/1299 (1.2%)	1/1027 (0.1%)	5/135 (3.7%)	5/73 (6.8%)	0/2 (0.0%)	4/71 (5.6%)	<0.001	<0.001	<0.001	0.300	0.520	0.747
**OUTCOMES**													
**Preeclampsia**	1280	16/1280 (1.3%)	9/1003 (0.9%)	4/134 (3.0%)	1/71 (1.4%)	0/2 (0.0%)	2/70 (2.9%)	0.033	0.665	0.116	0.486	0.959	0.551
**Gestational age at birth weeks**	1284	39.3 (1.7)	39.4 (1.7)	38.9 (1.7)	38.8 (1.5)	40.3 (1.6)	38.9 (1.7)	0.001	0.008	0.020	0.898	0.891	0.811
**Preterm delivery**	1284	68/1284 (5.3%)	47/1007 (4.7%)	9/134 (6.7%)	7/71 (9.9%)	0/2 (0.0%)	5/70 (7.1%)	0.302	0.053	0.350	0.425	0.909	0.563
**SVD**	1308	666/1308 (50.9%)	541/1027 (52.7%)	57/135 (42.2%)	35/73 (47.9%)	0/2 (0.0%)	33/71 (46.5%)	0.022	0.434	0.321	0.428	0.558	0.860
**Caesarean delivery**	1308	468/1308 (35.8%)	353/1027 (34.4%)	64/135 (47.4%)	23/73 (31.5%)	1/2 (50.0%)	27/71 (38.0%)	0.003	0.618	0.531	0.026	0.198	0.411
**Ventouse delivery**	1308	41/1308 (3.1%)	29/1027 (2.8%)	2/135 (1.5%)	5/73 (6.8%)	1/2 (50.0%)	4/71 (5.6%)	0.363	0.055	0.180	0.040	0.092	0.763
**Forceps delivery**	1308	107/1308 (8.2%)	82/1027 (8.0%)	11/135 (8.2%)	8/73 (11.0%)	0/2 (0.0%)	6/71 (8.5%)	0.947	0.370	0.889	0.502	0.940	0.612

**Neonatal sex (male)**	1275	646/1275 (50.7%)	508/999 (50.9%)	66/133 (49.6%)	35/71 (49.3%)	1/2 (50.0%)	36/70 (51.4%)	0.790	0.800	0.926	0.964	0.807	0.800
**Continued**	**n**	**ALL WOMEN n=1308**	**0: NGT n=1027**	**1: GDM-IR n=135**	**2: GDM-IS n=73**	**3: GDM-both n=2**	**4: GDM-neither n=71**	**p 0 vs 1**	**p 0 vs 2**	**p 0 vs 4**	**p 1 vs 2**	**p 1 vs 4**	**p 2 vs 4**
**Birthweight INTERGROWTH centile**	1272	65.1 (27.2)	63.9 (27.2)	73.0 (26.4)	67.7 (26.0)	61.2 (42.7)	63.9 (27.7)	<0.001	0.258	0.991	0.173	0.022	0.413
**Birthweight GROW centile**	1280	47.1 (29.6)	45.5 (29.0)	57.4 (32.3)	52.8 (29.5)	41.9 (32.9)	45.5 (30.2)	<0.001	0.045	0.988	0.312	0.010	0.149
**LGA INTERGROWTH**	1272	307/1272 (24.1%)	220/996 (22.1%)	51/133 (38.4%)	19/71 (26.8%)	1/2 (50.0%)	16/70 (22.9%)	<0.001	0.362	0.881	0.097	0.026	0.592
**LGA GROW**	1280	140/1280 (10.9%)	87/1003 (8.7%)	34/134 (25.4%)	10/71 (14.1%)	0/2 (0.0%)	9/70 (12.9%)	<0.001	0.124	0.236	0.061	0.037	0.831
**PPH**	1280	441/1280 (34.5%)	330/1003 (32.9%)	54/134 (40.3%)	27/71 (38.0%)	2/2 (100.0%)	28/70 (40.0%)	0.089	0.375	0.223	0.752	0.967	0.810
**Neonatal hypoglycaemi**a	1280	28/1280 (2.2%)	17/1003 (1.7%)	5/134 (3.7%)	3/71 (4.2%)	0/2 (0.0%)	3/70 (4.3%)	0.108	0.127	0.121	0.862	0.846	0.986
**Jaundice**	1280	82/1280 (6.4%)	55/1003 (5.5%)	17/134 (12.7%)	5/71 (7.0%)	0/2 (0.0%)	5/70 (7.1%)	0.001	0.581	0.559	0.214	0.226	0.981
**NICU admission**	1280	112/1280 (8.8%)	84/1003 (8.4%)	12/134 (9.0%)	8/71 (11.3%)	0/2 (0.0%)	8/70 (11.4%)	0.820	0.400	0.378	0.595	0.573	0.976

ESM Table 3: Comparison of multivariate prediction models for each pregnancy outcome. Three prediction models were evaluated for their ability to predict outcomes, with results presented as the Area Under the Receiver Operating Characteristic curve (AUROC). Model 1 includes maternal BMI, maternal age at OGTT, ethnicity, and parity. Model 2 extends Model 1 by adding fasting glucose. Model 3 builds on Model 2 and incorporates HOMA2-S and HOMA2-B scores.

	n	Model 1	Model 2	Model 3
**OUTCOMES**				
**Preeclampsia**	1280	0.70	0.70	0.75
**Preterm delivery**	1216	0.54	0.55	0.56
**SVD**	1282	0.63	0.63	0.63
**Caesarean delivery**	1282	0.60	0.61	0.61
**Ventouse delivery**	1282	0.68	0.69	0.74
**Forceps delivery**	1282	0.71	0.71	0.71
**LGA INTERGROWTH**	1272	0.62	0.64	0.64
**LGA GROW**	1280	0.57	0.61	0.62
**PPH**	1280	0.62	0.62	0.63
**Neonatal hypoglycaemia**	1280	0.58	0.59	0.61
**Jaundice**	1280	0.62	0.63	0.63
**NICU admission**	1280	0.59	0.59	0.59

ESM Figure 1: Comparison of categorisation of women using standard and enhanced processing.

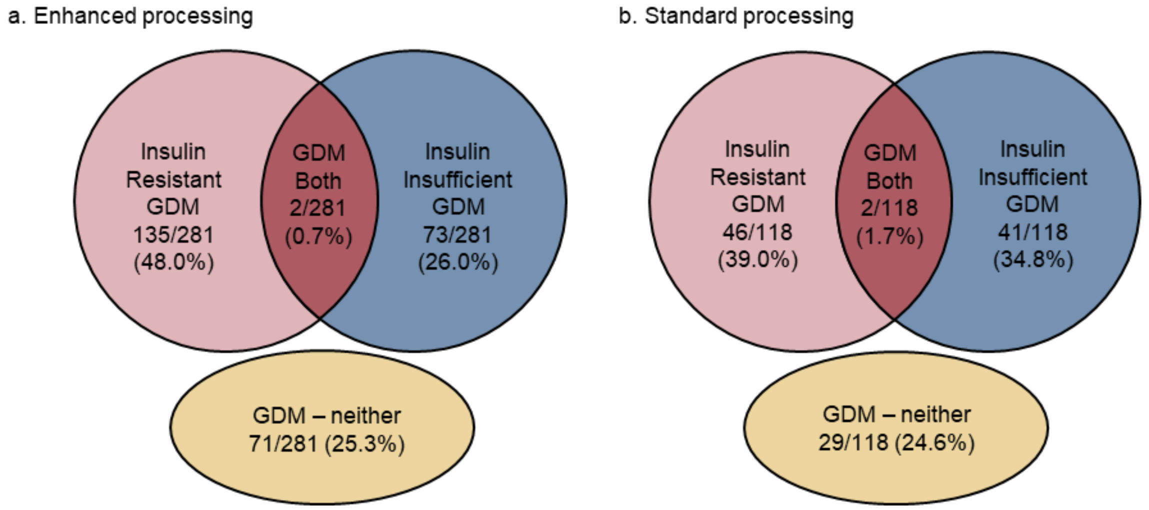

Supplement: Supplementary file 1 — ESM (PDF 219 KB) [file 125_2024_6334_MOESM1_ESM.pdf]
